# Analysis of the Influence of Fatigue Strength of Prototype Under Ballast Mats (UBMs) on the Effectiveness of Protection against Vibration Caused by Railway Traffic

**DOI:** 10.3390/ma14092125

**Published:** 2021-04-22

**Authors:** Cezary Kraśkiewicz, Artur Zbiciak, Anna Al Sabouni-Zawadzka, Michał Marczak

**Affiliations:** 1 Institute of Roads and Bridges, Faculty of Civil Engineering, Warsaw University of Technology, Al. Armii Ludowej 16, 00-637 Warsaw, Poland; a.zbiciak@il.pw.edu.pl; 2 Institute of Building Engineering, Faculty of Civil Engineering, Warsaw University of Technology, Al. Armii Ludowej 16, 00-637 Warsaw, Poland; a.sabouni@il.pw.edu.pl; 3 Faculty of Production Engineering, Warsaw University of Technology, Narbutta 85, 02-524 Warsaw, Poland; mim@meil.pw.edu.pl

**Keywords:** under ballast mats, vibration isolation, rheological model, fatigue strength, ballasted track structures

## Abstract

The present paper focuses on laboratory tests of fatigue strength of prototype under ballast mats (UBMs), carried out according to the procedure described in the new European standard EN 17282, which was released in October 2020. The mineral wool-based mat revealed significant differences in the values of static and dynamic characteristics, measured before and after the fatigue tests. The elastomeric mats based on recycled materials (SBR granulate and fibers) turned out to have had sufficient durability and effectiveness of protection against vibration caused by railway traffic. The values of static and dynamic bedding moduli, determined before and after the fatigue tests, were used to develop a viscoelastic rheological model of the vibration isolator with the use of fractional derivatives. Using this original model of the ballasted track system with four degrees of freedom, a significant influence of cyclic loading on the level of vibration suppression (insertion loss factor) was demonstrated. The analytical model confirmed that the mats with variations of bedding moduli exceeding 10% should not be used as resilient elements.

## 1. Introduction

Under ballast mats (UBMs) have been used since the 1960s [1]. These resilient elements have two functions: reduction of vibrations caused by railway traffic and protection of the ballast layer against fast degradation due to the abrasion and breakage of the ballast particles.

An effort has been made to find efficient solutions that would enhance the effectiveness of vibration isolation of various resilient elements applied in track systems. One of the solutions proposed in the literature is the application of rubber-based mats. Rubber-based mats have a wide range of applications as a common vibration isolation material in various track systems. They can improve the deformation behaviors of both ballasted and ballastless track systems. Zheng et al. [2] analyzed rubber-based mats that were applied in ballastless tracks laid on bridges to improve their service performance. Results of static tests performed on full-scale ballastless tracks with various isolation layers were discussed. Sheng et al. [3] investigated how the rubber isolation layers influence the behavior of the ballastless track. Results of experimental tests reveal that the rubber layers exhibit nonlinear behavior that becomes apparent with the increased loading. Another possible application of rubber mats is their use in transition zones between two different slab tracks in high-speed railway [4]. Such mats can solve the transition problem by gradually changing the vertical track stiffness. Resilient mats made of recycled rubber were also discussed by Lapcik et al. [5], where the authors measured the dynamic stiffness of the rubber mats used in railway track structures. Horníček et al. [6] investigated the long-term behavior of antivibration mats made of recycled rubber. Changes in elastic properties of the mats after their exposure to cyclic loading were analyzed and resistance of the mats was determined using the German methodology that was modified for use on Czech railway tracks.

The rubber can be used to manufacture resilient elements such as UBMs and other vibration-isolating mats, but it can also be applied as a material of the sleepers [7]. Zeng et al. [7] discussed results of full-scale laboratory tests performed on a ballasted track structure with rubber composite sleepers, which proved that the application of rubber sleepers leads to better vibration attenuation than in the case of concrete sleepers. The ballasted track with rubber composite sleepers exhibits a significant reduction effect on the ground.

There are many possible methods for analyzing and assessing the vibration isolation function of UBMs and other resilient elements. Wettschureck et al. [8] proposed a one-degree-of-freedom (1DoF) model of the track structure with a UBM. A similar approach was applied by Hansona et al. [9], where the authors discussed differences between the expected level of the vibration suppression calculated with the use of a 1DoF analytical model of the track with a UBM and the values measured on the real track in a high range of frequencies (over 100 Hz). Choi et al. [10] showed results of theoretical, experimental and analytical analyses that were performed in order to predict and evaluate the dynamic behavior of a ballasted track. They developed a 2DoF dynamic model of the track that allowed them to define the rail pad and ballast stiffness ranges based on the values obtained from laboratory tests. Analysis of these works leads to the conclusion that it is better to use models with more degrees of freedom, as they give more accurate results.

An interesting approach was discussed in [11], where Zakeri et al. analyzed the ground-borne vibrations caused by railway traffic. They proposed an inexpensive and efficient method of controlling these vibrations by adjusting the geometry and weight of building foundation. Qu et al. [12] investigated the vibration mitigation effects of the ballasted ladder track with various elastic elements. The authors used a method that combines the multi-body dynamics (MBD) and the finite element method (FEM). The basics of the joint use of both methods in structural analysis were explained by Al Sabouni-Zawadzka et al. [13], where this approach was applied to deployable systems. Zhao et al. [14] focused on the evaluation method of the vibration reduction effect taking into consideration the real load- and frequency-dependent stiffness of under slab mats (USMs). The authors tested the static nonlinearity and dynamic properties of three USMs and applied a modified fractional derivative Poynting–Thomson model to describe the preload and frequency dependence.

Based on the performed literature study, it can be stated that UBMs are elements with a wide range of possible applications, which are worth a more thorough analysis. However, the research presented in the literature is not comprehensive and needs to be extended, especially in the light of new standardization and the lack of sufficient data on fatigue strength analysis.

There are many methods that can be used for improving the fatigue properties of various materials. Panin et al. [15] studied a five-stage helical rolling (HR) as a means to increase the fatigue life of the high-strength low-alloyed steel. It was shown that the five-stage HR resulted in an increase in the steel fatigue life by more than 3.5 times under cyclic tension. Ivanov et al. [16] focused on the fatigue life of silumin treated with a high-intensity pulsed electron beam. They determined an optimum irradiation mode that allows one to increase the fatigue life of this material by a factor of up to 3.5.

The main aim of this paper is the analysis of the influence of fatigue strength of prototype UBMs on the effectiveness of protection against vibration caused by railway traffic. The authors present novel results obtained in the fatigue tests that were conducted according to the requirements of the newly released (October 2020) standard EN 17282 [17]. Two types of the mats materials were considered: mineral wool and recycled rubber. As it was proved by Kraśkiewicz et al. [18] for under sleeper pads (USPs), fatigue strength of the resilient elements is an important factor that affects the durability of the railway track structure and the effectiveness of vibration isolation.

Results obtained in laboratory fatigue tests were used in the rheological model of the ballasted track structure with a UBM. The original 4DoF model was presented in the previously published works of the authors [19]. Using this model, diagrams of transmissibility and insertion loss function were prepared.

## 2. Laboratory Testing Procedures and Requirements for the Fatigue Strength of UBMs

### 2.1. Fatigue Strength Testing Procedures

According to the newly released standard EN 17282 [17], there are three tests that need to be performed while determining the service life of a UBM:

- Fatigue test on a UBM in a steel box filled with ballast;
- Fatigue test on a UBM with a geometric ballast plate (GBP) (for UBMs used as vibration isolators);
- Test of resistance to severe environmental conditions, such as water, high temperatures and freeze–thaw resistance.

Fatigue strength and resistance to severe environmental conditions of vibration isolators are very important factors that affect the durability of the railway track structure and the effectiveness of vibration isolation. The durability of UBMs can be assessed by testing their mechanical fatigue strength and the influence of long-term dynamic loads on the variability of certain parameters. The test consists of applying a dynamic load to the UBM sample, which simulates service loads in laboratory conditions.

The fatigue test on a UBM in a ballast box, according to EN 17282 [17], should be performed on the UBM sample consisting of two identical parts with the total dimensions of 1000 mm × 1000 mm (including their connection). The most important change in relation to the previous regulations concerns the number of load cycles—while the German standard DIN 45673-5 [20] required 12.5 million cycles, the new European standard EN 17282 [17] requires only 2.5 million load cycles. Moreover, according to DIN 45673-5 [20], variation of the static bedding modulus had to be determined and EN 17282 [17] does not require it. A positive assessment of the fatigue strength of a UBM in the case of the test in a ballast box includes only a confirmation of the lack of visible mechanical damage (perforation, cracking or other damage).

In the fatigue test with GBP, according to EN 17282 [17], apart from the visual inspection, a range of variations of static and dynamic (for 5 Hz) bedding moduli under 3 million load cycles has to be determined. This test makes it possible to assess the influence of cyclic loading on the effectiveness of vibration isolation.

Assessment of the durability of UBMs—evaluated based on their fatigue strength—is important from the point of view of the isolation effectiveness (isolating properties of UBMs). In the ballasted track structure, a replacement of UBMs would result in a major repair of the track structure, that is, removal and reconstruction of all the structural elements (rails, rail fastenings, sleepers and ballast). Therefore, durability is crucial for infrastructure managers, who cannot allow themselves to use UBMs with durability that is lower than the durability of other structural elements.

Selection of a UBM during the design process has to include its fatigue strength. Even if other properties of the mat are satisfactory, neglecting the durability parameter may lead to a fast increase of the bedding moduli under service loads, which may affect one of its main functions—the required effectiveness of vibration isolation.

### 2.2. Geometric Ballast Plate

The geometric ballast plate (GBP) that was used in the fatigue tests of UBMs was manufactured at the Warsaw University of Technology, in accordance with the requirements of EN 17282 [17]. The main function of the GBP is a simulation of the interaction between the ballast grains and the mat during the laboratory test, which is far more reliable than in the case of a flat steel plate (as required by DIN 45673-5 [20]). The surface of the GBP is formed with three types of pyramid elements, which accurately simulate the point contact of the UBM with sharp edges of the ballast particles of different sizes and shapes. The assurance of the point contact is especially important in the fatigue tests because the tested mat is more prone to permanent damage (as in the real ballast structure) as opposed to the test with a flat plate.

**Figure 1:**
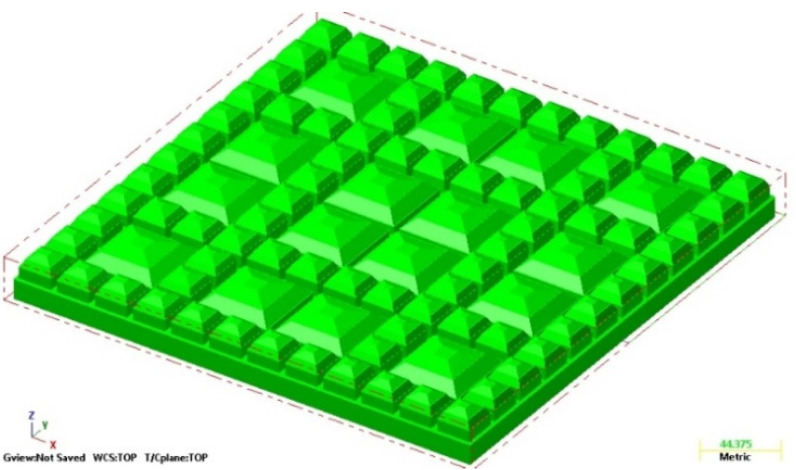
Vector model of GBP for the UBM tests according to EN 17282 [17].

**Figure 2:**
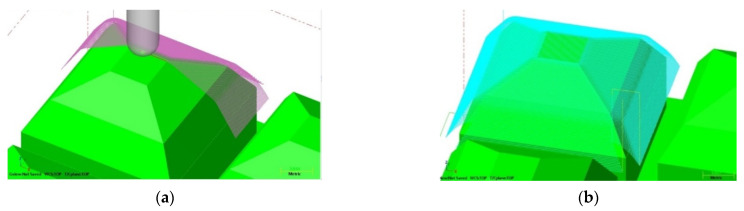
Simulation of the milling of GBP: (**a**) general view; (**b**) milling path.

**Figure 3:**
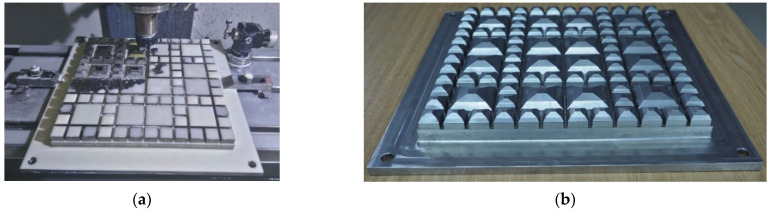
Geometric ballast plate for the UBM tests: (**a**) during the metalworking on the three-axis CNC HAAS MiniMill milling machine; (**b**) ready to use.

In order to manufacture the geometric ballast plate, a vector 3D model in the Creo Parametrics 2.0 (PTC Inc., Boston, MA, USA) environment was prepared, which was afterward imported to the Mastercam X5 (CNC Software Inc., Tolland, CT, USA) software (Figure 1). Then, a metalworking technology was developed (Figure 2), and the plate was manufactured on the three-axis CNC HAAS MiniMill milling machine (Figure 3).

The plate was made of steel 304L, which is an austenitic nickel-chrome steel with good resistance to corrosion (Table 1). The dimensions of the GBP are consistent with the requirements of EN 17282 [17]: 300 mm × 300 mm × 40 mm.

### 2.3. Requirements for the Fatigue Strength of UBMs

The authors reviewed the requirements of foreign railway infrastructure managers and prepared a tabular comparison of the limiting values for the parameters that are particularly important from the point of view of the proper selection of UBMs, with regard to their durability (Table 2). In the last column of Table 2, the authors include their own initial recommendations toward the considered requirements.

Based on the gathered data and the requirements of EN 17282 [17], the authors formulated initial recommendations that can be used as design assumptions in the laboratory tests aimed at material preselection (limited to 500,000 load cycles) of prototype UBMs—as presented in Section 3 of this paper:

- No perforations, cracks, visible damage during and after the test;
- Variation of the static bedding modulus Δ*C*_stat_ ≤ 10%;
- Variation of the dynamic bedding modulus Δ*C*_dyn_ ≤ 10%.

## 3. Laboratory Tests of Fatigue Strength

### 3.1. Tests Description

**Figure 4:**
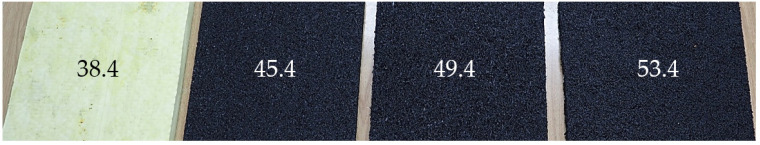
UBM samples before fatigue tests: 38.4 (mineral wool), 45.4, 49.4 and 53.4 (SBR).

Fatigue tests were performed on four samples of UBMs (Figure 4):

- 38.4—mineral wool sample, thickness 35 mm;
- 45.4—styrene-butadiene rubber (SBR) sample, thickness 20 mm, density 500 kg/m^3^;
- 49.4—styrene-butadiene rubber (SBR) sample, thickness 20 mm, density 600 kg/m^3^;
- 53.4—styrene-butadiene rubber (SBR) sample, thickness 20 mm, density 700 kg/m^3^.

The samples had dimensions of 300 mm × 300 mm. The material used for rubber-based mats can be classified as an elastomer. This type of material does not lose its structural continuity when submitted to tension or compression; it can undergo large deformations and regains its initial shape after removal of the load. The mineral wool, on the other hand, cannot be classified as an elastomer.

Mineral wool mats have already been applied in some ballastless and ballasted track structures, mainly in Sweden, Norway and Denmark. The authors have decided to investigate the prototype UBMs based on mineral wool in the same conditions as SBR-based mats in order to compare their fatigue properties and durability.

The tests were carried out according to EN 17282 [17] with some changes in the testing procedure, which were aimed at a more thorough investigation of static and dynamic parameters of UBMs under cyclic loading. Due to the fact that the main aim of the performed tests was to preselect the prototype mats before the next research phase, 500,000 load cycles were used (applied by a pulsator that was controlled with the value of adopted load ranges). The reduced number of load cycles (500,000 instead of 3 million as required by EN 17282 [17]) was motivated with a preliminary character of the tests—the adopted value was enough to preselect the analyzed mats, indicate the ones that should be tested further and reject the mats that could not be defined as promising, durable vibration isolators. The values of static and dynamic bedding moduli, with the dynamic load frequency of 5 Hz, were measured every 125,000 load cycles. The dynamic bedding modulus test was extended in respect to the requirements of EN 17282 [17]—additional frequencies were considered, and a displacement-controlled test was performed.

According to EN 17282 [17], samples were tested at room temperature (23 ± 5 °C) using a GBP. Before the test, a visual inspection of samples was performed in order to find potential damage caused by their transportation, storage or assembly at the test stand.

**Figure 5:**
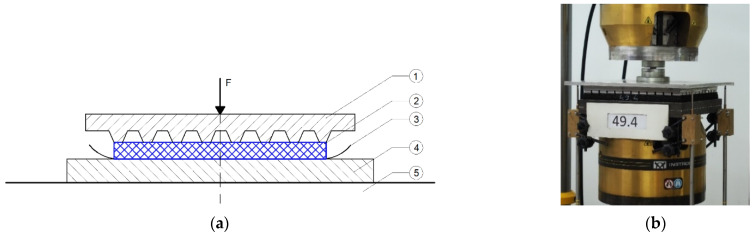
Fatigue test on UBM with GBP: (**a**) test scheme: 1—GBP, 2—UBM sample, 3—abrasive paper, 4—steel plate, 5—rigid support (scheme according to EN 17282 [17]); (**b**) UBM 49.4 sample in the laboratory test stand.

Figure 5 depicts a scheme of the fatigue test on the UBM. During the test, the following parameters (according to EN 17282 [17] for track category TC3) were determined:

- Static bedding moduli: *C*_stat_ and *C*_tend_;
- Dynamic bedding modulus *C*_dyn_ at frequencies 1, 2, 3, 5, 10, 15 and 20 Hz, using the load-controlled method, with the load range 1.8 kN to 9.0 kN;
- Dynamic bedding modulus *C*_H_ at frequencies 8, 10, 12.5, 16, 20 and 24 Hz, using the displacement-controlled method, with the initial static load of 9.0 kN.

The same values were determined again after 1 to 2 weeks after the fatigue test. Definitions of static and dynamic bedding moduli, together with the detailed descriptions of the tests, can be found in previous works of the authors [28,29].

In the next step, the samples were submitted to 500,000 load cycles with the value from *F*_min_ = 1.8 kN to *F*_test_ = 9.0 kN, after which the parameters defined above were determined once again using both the load- and displacement-controlled methods. The static and dynamic (at 5 Hz) bedding moduli were tested further every 125,000 load cycles, that is, after 125,000, 250,000 and 375,000 load cycles.

After the tests, a visual inspection of the samples was performed, which was aimed at finding potential damage arisen during the tests, such as perforation, cracking or other damage. If any damage was found, the samples were qualified as not fulfilling the requirements for the fatigue strength and rejected.

Variations of the determined values of bedding moduli were calculated using the following formulas:

(1) $$\Delta C_{\mathrm{stat}}=\frac{C_{\mathrm{stat},\mathrm{post}}-C_{\mathrm{stat},\mathrm{pre}}}{C_{\mathrm{stat},\mathrm{pre}}}\cdot 100\;\left[\%\right],$$

(2) $$\Delta C_{\mathrm{tend}}=\frac{C_{\mathrm{tend},\mathrm{post}}-C_{\mathrm{tend},\mathrm{pre}}}{C_{\mathrm{tend},\mathrm{pre}}}\cdot 100\;\left[\%\right],$$

(3) $$\Delta C_{\mathrm{dyn},5\mathrm{Hz}}=\frac{C_{\mathrm{dyn},5\mathrm{Hz},\mathrm{post}}-C_{\mathrm{dyn},5\mathrm{Hz},\mathrm{pre}}}{C_{\mathrm{dyn},5\mathrm{Hz},\mathrm{pre}}}\cdot 100\;\left[\%\right],$$

where:

$\Delta C_{\mathrm{stat}}$—variation of the static bedding modulus determined at the load range 0.02÷0.10 N/mm^2^,

$C_{\mathrm{stat},\mathrm{post}}$—value of the static bedding modulus determined at the load range 0.02÷0.10 N/mm^2^, measured after the test,

$C_{\mathrm{stat},\mathrm{pre}}$—value of the static bedding modulus determined at the load range 0.02÷0.10 N/mm^2^, measured before the test,

$\Delta C_{\mathrm{tend}}$—variation of the static bedding modulus determined at the load range 0.02÷0.20 N/mm^2^,

$C_{\mathrm{tend},\mathrm{post}}$—value of the static bedding modulus determined at the load range 0.02÷0.20 N/mm^2^, measured after the test,

$C_{\mathrm{tend},\mathrm{pre}}$—value of the static bedding modulus determined at the load range 0.02÷0.20 N/mm^2^, measured before the test,

$\Delta C_{\mathrm{dyn},5\mathrm{Hz}}$—variation of the dynamic bedding modulus determined in the load-controlled test at the frequency of 5 Hz,

$C_{\mathrm{dyn},5\mathrm{Hz},\mathrm{post}}$—value of the dynamic bedding modulus determined in the load-controlled test at the frequency of 5 Hz, measured after the test,

$C_{\mathrm{dyn},5\mathrm{Hz},\mathrm{pre}}$—value of the dynamic bedding modulus determined in the load-controlled test at the frequency of 5 Hz, measured before the test.

The test stand consisted of a universal testing machine INSTRON 8802 (Figure 5) with two steel plates: lower (support plate) with the dimensions of 320 mm × 320 mm and upper GBP. During the tests displacements in four points were measured, using displacement inductive sensors (WA-T type by HBM, Hottinger Baldwin Messtechnik GmbH, Darmstadt, Germany) together with HBM’s Spider8 data acquisition and signal conditioning system and dedicated software—Catman AP (Version 3.4).

### 3.2. Results of Laboratory Tests

**Figure 6:**
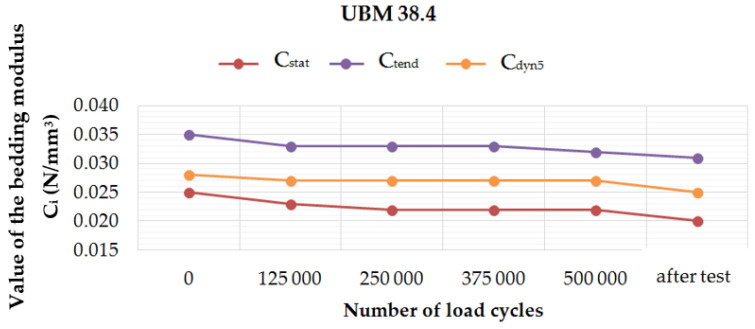
Variation of the static and dynamic bedding moduli (5 Hz) for sample no. 38.4, determined during and after the fatigue test.

**Figure 7:**
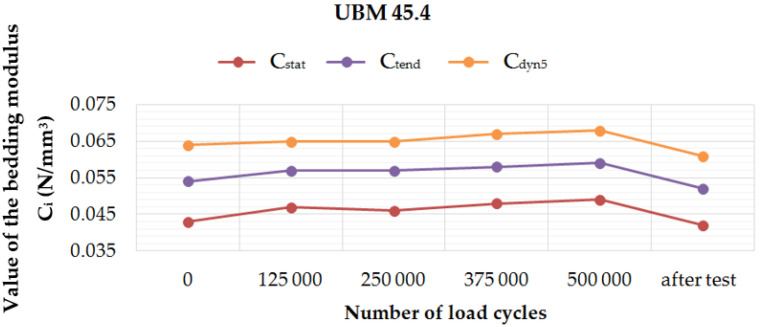
Variation of the static and dynamic bedding moduli (5 Hz) for sample no. 45.4, determined during and after the fatigue test.

**Figure 8:**
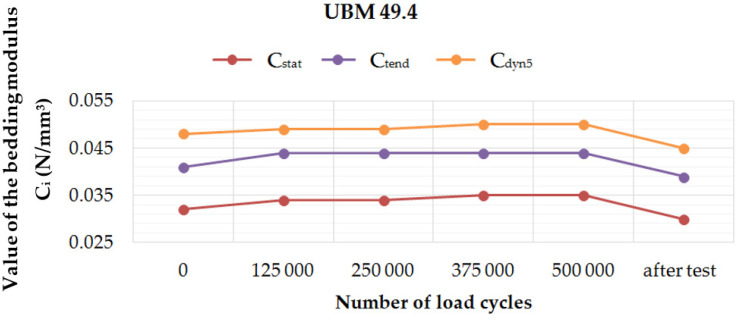
Variation of the static and dynamic bedding moduli (5 Hz) for sample no. 49.4, determined during and after the fatigue test.

**Figure 9:**
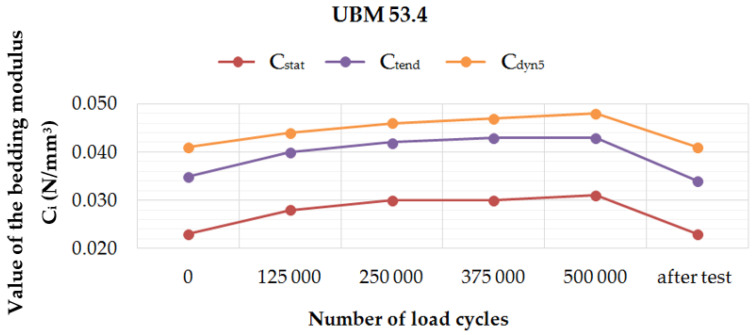
Variation of the static and dynamic bedding moduli (5 Hz) for sample no. 53.4, determined during and after the fatigue test.

Figure 6, Figure 7, Figure 8 and Figure 9 present diagrams of variations of static (*C*_stat_ and *C*_tend_) and dynamic (*C*_dyn5_) bedding moduli at 5 Hz, during and after the fatigue tests performed on four UBM samples. It can be noticed that for UBM 38.4 the values of all determined parameters decreased with the applied load cycles and after two weeks from the test, which means that the material stiffened. It points to a high material heterogeneity of mineral wool and possible damage of the material macrostructure caused by the cyclic dynamic loading. In the case of rubber-based samples, UBM 45.4, 49.4 and 53.4, the values of all tested parameters increased during the test, reached maximum after 500,000 load cycles and decreased two weeks after the test. It indicates the dynamic stiffening effect that is characteristic of elastomers. The bedding moduli grow during the test, and the elastic recovery of the samples can be observed after the test.

**Figure 10:**
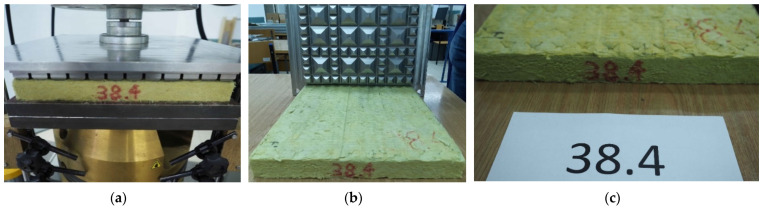
Visual inspection of UBM 38.4 according to EN 17282 [17]: (**a**) first damage—material chipping during the initial series of 100,000 load cycles; (**b**) sample after 500,000 load cycles (visible print from GBP on the mat surface); (**c**) sample after 500,000 load cycles with visible damage.

After the fatigue tests (after 500,000 load cycles), all samples were submitted to visual inspection with an unaided eye, according to EN 17282 Annex E: “After the end of fatigue test and the end of bedding modulus test, the UBM shall be visually inspected in order to look for evidence of damage (visual assessment of evidence of perforation, cracking or other damage)” [17]. In the mineral wool sample UBM 38.4, much damage (chipping and cracking) and permanent deformations (print from the GBP on the upper surface of the mat) were identified (Figure 10).

**Figure 11:**
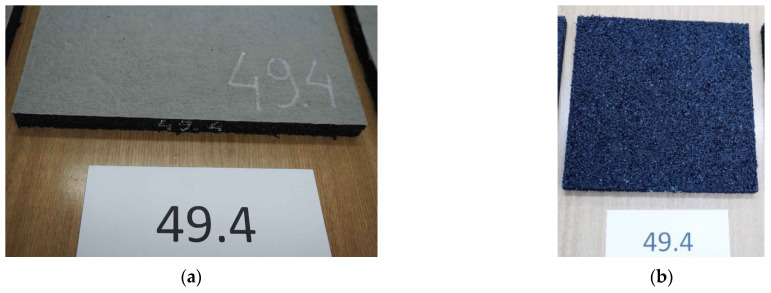
Visual inspection of UBM 49.4 according to EN 17282 [17]: (**a**) undamaged upper surface of the mat with a geotextile protection layer (no print from GBP); (**b**) undamaged lower surface of the mat without the protection layer (visible inner structure of the rubber-based mat).

The chipping and changes in the material macrostructure were visible already in the first series of 100,000 load cycles. In rubber-based samples UBM 45.4, 49.4 and 53.4, no damage was identified. Figure 11 presents photos of the upper and lower surfaces of the UBM 49.4 with no visible damage.

Table 3 presents results of fatigue tests performed on prototype UBM samples, which were aimed at classifying the mats in terms of their durability (recommendations of the requirements proposed by the authors in Section 2) and the initial preselection of the mats for further tests. The analyzed rubber mats were based on recycled SBR granulate and fibers, joined together with a polyurethane adhesive. Due to some technological limitations, they may have differed not only in density but also in the amount of adhesive and the proportion of granulate and fibers used. A slight variation of these parameters (apart from the density) may have influenced the fatigue properties of considered samples. The presented tests indicate that the optimal formula was selected for UBM 45.4, where more SBR fibers were used than in the two remaining rubber-based mats, and this increased the invariability of the bedding moduli.

Additionally, going behind the requirements of EN 17282 [17], the values of dynamic bedding modulus using the displacement-controlled method were determined. This parameter is used in the analyses of the vibration suppression level (see Section 4).

**Figure 12:**
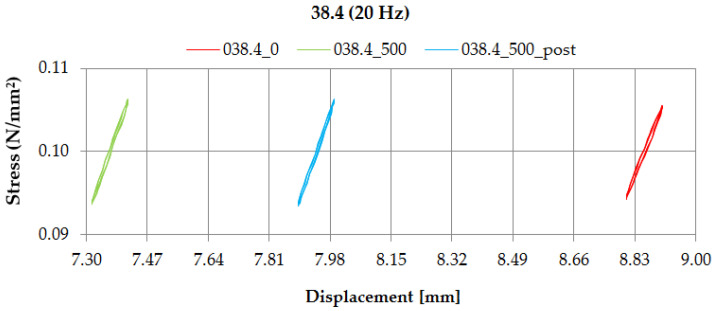
Dynamic characteristics of UBM 38.4 determined in the displacement-controlled test at 20 Hz.

**Figure 13:**
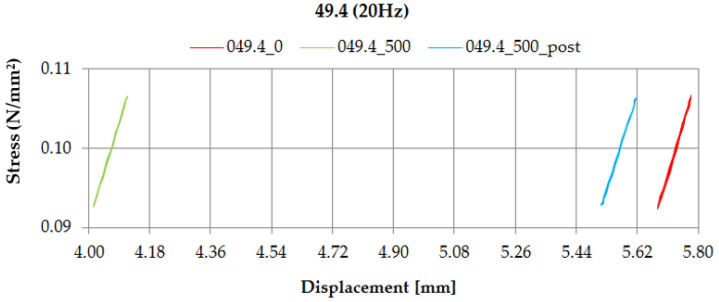
Dynamic characteristics of UBM 49.4 determined in the displacement-controlled test at 20 Hz.

Figure 12 and Figure 13 depict diagrams of dynamic characteristics (stress-displacement relations) of samples UBM 38.4 and 49.4, determined in the dynamic bedding moduli (*C*_H_) tests at the frequency of 20 Hz, using the displacement-controlled method. Red lines correspond to the values determined before the fatigue test (designated as “0”), green—values determined after 500,000 load cycles (designated as “500”) and blue—values determined two weeks after the fatigue test (designated as “500_post”). 

### 3.3. Discussion of Results

Performed tests allowed the authors to formulate the following conclusions:

- Mineral wool sample UBM 38.4 did not exhibit a sufficient fatigue strength in the tests aimed at the preselection of the mats because the variations of static and dynamic bedding moduli exceeded the limiting value of 10% (the value recommended by the authors for the tests performed up to 500,000 load cycles). A possible reason may be the fact that the material macrostructure of the sample was damaged, which caused the decrease of tested parameters and the sample degradation after the application of cyclic loading;
- During the fatigue tests of rubber-based UBM samples, a significant dynamic stiffening effect was identified. The values of dynamic bedding modulus increased and the samples did not undergo a full relaxation between successive load cycles;
- Rubber-based samples UBM 45.4, 49.4 and 53.4 exhibited sufficient fatigue strength and therefore were selected for further tests. There was no visible damage on the surfaces of these samples after the tests, no material chipping, perforations or cracking. A slight decrease in the values of tested parameters (≤10%) is acceptable and may have resulted from some fine damage of the samples’ macrostructure. The samples were made of SBR granulate and fibers glued with a polyurethane adhesive. During the fatigue test, the connection between the granulate and fibers may have been damaged, which resulted in a small decrease in the measured parameters;
- In the case of mineral wool-based UBM 38.4, the value of *C*_stat_ decreased by 20.0%, *C*_dyn5_ decreased by 10.7% and *C*_H_ increased by 9.8 ÷ 15.1%—depending on the load frequency. The obtained results—especially large variations of the bedding moduli determined before and after the tests—point to significant heterogeneity of the material, which means that the mat is not suitable for the assumed application;
- In the case of rubber-based UBM 49.4, the value of *C*_stat_ decreased by 6.3%, *C*_dyn5_ decreased by 6.4% and *C*_H_ decreased by 4.9 ÷ 8.4%—depending on the load frequency. The obtained results point to homogeneity of the material;
- The material of the mat has a significant influence on its fatigue strength—elastomeric materials that are produced from the recycled old tires (based on SBR granulate and fibers) exhibit satisfactory characteristics and are suitable for the assumed application.

## 4. Mechanical Model of the Structure

**Figure 14:**
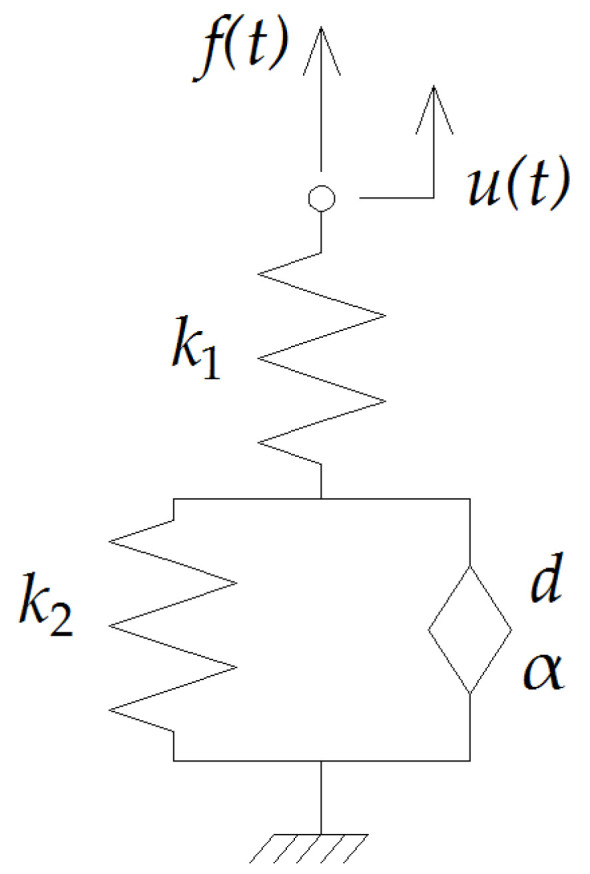
Fractional Zener model representing rheological properties of UBM.

In order to describe rheological properties of UBMs, the Fractional Zener (FZ) model was used (see Figure 14). The model is composed of two springs $k_{1}$ and $k_{2}$ and a fractional element (spring-pot) defined by two parameters d and α. The FZ model contains a non-classical viscoelastic element whose constitutive properties are defined by fractional derivatives [30]. It can be proved that, in the case of the FZ model, the fractional-order differential equation (FDE) describing the relationship between the force f(t) and the displacement u(t) is as follows:

(4) $$f\left(t\right)+\frac{\tau k_{\mathrm{eq}}}{k_{1}}D^{\alpha }f\left(t\right)=k_{\mathrm{eq}}u\left(t\right)+\tau k_{\mathrm{eq}}D^{\alpha }u\left(t\right),$$

where:

$k_{\mathrm{eq}}=\frac{k_{1}k_{2}}{k_{1}+k_{2}}$ and $\tau =\frac{d}{k_{2}}$.

The following definition of the fractional derivative of the order α∈(0,1) for a function z(t) can be applied to Equation (4) [31]:

(5) $$D^{\alpha }z\left(t\right)=\frac{z\left(0\right)}{\Gamma \left(1-\alpha \right)t^{\alpha }}+\frac{1}{\Gamma \left(1-\alpha \right)}\int_{0}^{t}\frac{\dot{z}\left(\xi \right)}{{\left(t-\xi \right)}^{\alpha }}d\xi ,$$

where:

Γ(⋅) denotes the gamma function.

Applying Laplace transforms to Equation (4) leads to the following equations:

(6) $$f^{*}\left(s\right)=k^{*}\left(s\right)\;u^{*}\left(s\right),$$

(7) $$k^{*}\left(s\right)=\frac{k_{1}\left(k_{2}+ds^{\alpha }\right)}{k_{1}+k_{2}+ds^{\alpha }}$$

where:

$f^{*}\left(s\right)=L\left\{f\left(t\right)\right\}$ and $u^{*}\left(s\right)=L\left\{u\left(t\right)\right\}$ are the Laplace transforms of the force and the displacement, respectively.

The complex stiffness modulus $k^{*}\left(i\omega \right)$ of the FZ model can be obtained directly from Equation (7) when substituting s by iω, i.e.,

(8) $$k^{*}\left(i\omega \right)=k^{*}\left(s\right)\;{|}_{s=i\omega }$$

where:

ω denotes the frequency of excitation and i represents the imaginary unit satisfying the equation $i^{2}=-1$.

The complex stiffness modulus constitutes the amplitude of the harmonic steady-state force response $f^{*}\left(i\omega \right)=k^{*}\left(i\omega \right)e^{i\omega t}$ exited by the complex displacement $u^{*}\left(i\omega \right)=e^{i\omega t}$. Moreover, the frequency response of any linear system can be characterized by the magnitude $\left|k^{*}\left(i\omega \right)\right|$ called the dynamic stiffness modulus $k_{\mathrm{dyn}}\left(\omega \right)$.

The authors decided to present the results of the analytical analysis only for the mineral wool-based mat UBM 38.4, which exhibited the lowest fatigue strength in the experimental tests. In the case of this mat, the variations of the static and dynamic parameters determined in the laboratory exceeded the limiting values of 10% that were recommended by the authors in Section 2.3. The authors wanted to prove that such variations are not acceptable, as they may affect the effectiveness of vibration suppression during the whole exploitation period. It should be assumed that the loss of vibration isolation properties would be even higher in real service conditions, as there are more load cycles than the tested 500,000. Moreover, the mat would be faster damaged as a result of the contact with real ballast grains.

**Figure 15:**
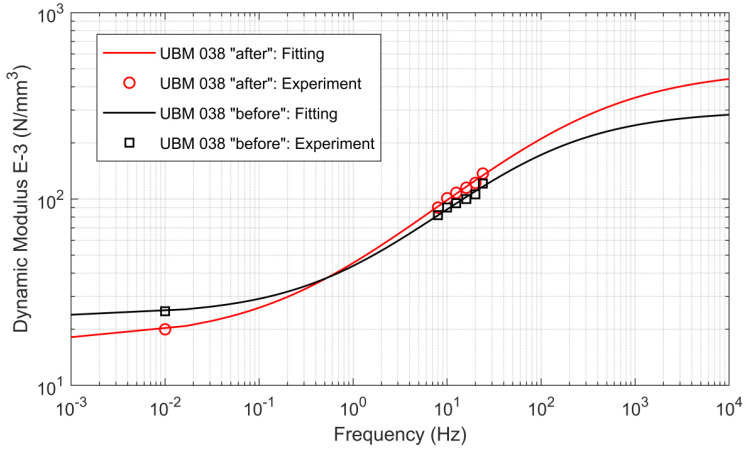
Curve fitting results for the dynamic bedding modulus of UBM 38.4.

Figure 15 presents results of the FZ model curve fitting procedure performed for the dynamic bedding modulus of UBM 38.4 using the results of laboratory tests of UBM (before and after the fatigue test). The theoretical model is consistent with the experimental tests’ results.

**Figure 16:**
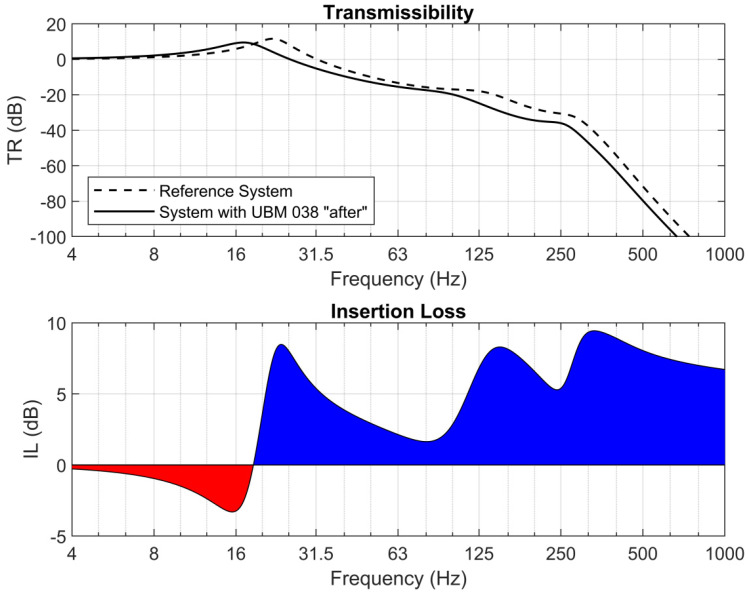
Transmissibility and insertion loss—reference system and system with UBM 38.4.

Parameters of the FZ model that were obtained in this analysis were used in the 4DoF model, which describes the behavior of the ballasted track structure. The original 4DoF model was presented in the previously published works of the authors [19]. Using this model, diagrams of transmissibility and insertion loss (IL) function were prepared. Diagrams presented in Figure 16 correspond to the UBM properties that were measured after the fatigue tests. The values of IL obtained for the cases “before” and “after” the tests are gathered in Table 4. Analysis of these values shows a significant decrease of IL for the case “after” at high frequencies, which can result in an increased vibration transmission and enhance the secondary structure-borne noise.

Analysis of the mechanical model of the structure indicated that the low fatigue strength of the UBM, measured as a variation of the static and dynamic bedding modulus, results in a significant decrease in the effectiveness of vibration isolation. Analytical results confirm that the mineral wool-based mat UBM 38.4 is not suitable for the assumed application and should be rejected at this stage of the tests.

## 5. Conclusions

The current work focused on the analysis of the influence of fatigue strength of prototype under ballast mats (UBMs) on the effectiveness of protection against vibration caused by railway traffic. The authors presented novel results obtained in the fatigue tests that were performed in accordance with the requirements of the new standard EN 17282 [17], released in October 2020. Four samples of UBMs were tested: three of them were made of styrene-butadiene rubber (SBR), and one was made of mineral wool.

The prototype mineral wool sample did not exhibit a sufficient fatigue strength, as the variations of static and dynamic bedding moduli exceeded the limiting value of 10%. The obtained results point to significant heterogeneity of the material, which means that the mat is not suitable for the assumed application as a vibration isolator. Rubber-based samples exhibited sufficient fatigue strength and therefore were indicated for further tests. There was no visible damage on the surfaces of these samples after the tests, no material chipping, perforations or cracking.

The mineral wool-based mat was then analyzed analytically using a 4DoF rheological model of the ballasted track structure with a UBM, developed by the authors. Results obtained from the mechanical model of the structure prove that the low fatigue strength of the UBM, determined as a variation of the static and dynamic bedding modulus, resulted in a significant decrease in the effectiveness of vibration isolation. An analytical model confirmed that the prototype mineral wool-based UBM, with its variations of the static bedding modulus at the level of 20% and the dynamic bedding modulus exceeding 10%, should not be used as a resilient element aimed at isolation of the vibrations caused by railway traffic.

## Figures and Tables

**Table 1:** Designation, parameters and chemical composition of 304L stainless steel [21].

Standard/Parameters	Mark/Value	Chemical Composition (%)	
Grade AISI	304L	C	0.03
Grade DIN	1.4307	Mn	2
Grade BS	304S11	Si	≤1
Tensile strength *R*_m min_ (MPa)	620	P	0.045
Proof stress *R*_0.2 min_ (MPa)	310	S	0.03
Relative elongation *A*_5_ (%)	45	Cr	18–20
		Ni	10.5
		N	0.1

**Table 2:** Requirements for fatigue strength of UBMs according to the regulations in various countries and the initial recommendation of the authors.

Properties		Germany [22]	Belgium [23,24]	Switzerland [1,25]	France [1,26]	Italy [1,27]	Authors’ Recommendations
**Fatigue strength (ballast box)**	**visual inspection**	no damage	no damage	no damage	no damage	-	**no damage**
	**Δ*C*_stat_**	≤10%	≤20%	≤10%	-	-	**≤20%**
	**Δ*C*_dyn_ (5 Hz)**	-	≤20%	-	-	-	**≤20%**
**Fatigue strength (GBP, 3 million cycles)**	**visual inspection**	-	-	-	-	no damage	**no damage**
	**Δ*C*_stat_**	-	-	-	-	≤20%	**≤15%**
	**Δ*C*_dyn_ (5 Hz)**	-	-	-	-	-	**≤15%**

**Table 3:** Requirements for fatigue strength of UBMs according to the regulations in various countries and the initial recommendation of the authors.

Sample No.	No Perforations, Cracking or Other Visible Damage	Δ*C*_stat_ (%)	Δ*C*_dyn5_ (%)	Requirements Fulfillment
38.4	−	−20.0	−10.7	−
45.4	+	−2.3	−4.7	+
49.4	+	−6.3	−6.4	+
53.4	+	0.0	0.0	+

**Table 4:** Insertion loss values obtained for UBM 38.4 from the 4DoF mechanical model in 1/3 octave bands, before and after the fatigue test.

**UBM 38.4**	**Frequencies in 1/3 Octave Bands *f* (Hz)**					
	31.5	63	125	250	500	1000
	**IL (dB)**					
**before**	5.976	2.416	7.872	6.431	9.675	8.414
**after**	5.389	2.143	6.814	5.398	8.049	6.719

## Data Availability

The data presented in this study are available on request from the corresponding author. The data are not publicly available due to the restrictions of the realized project and the authors’ will to patent some of the invented solutions.
